# The Principles and Practice of Surgery

**Published:** 1884-02

**Authors:** 


**The Principles and Practice of Surgery**: Being a Treatise on Surgical Diseases and Injuries. By D. HAYES AGNEW, M. D., LL. D., Professor of Surgery in the Medical Department of the University of Pennsylvania. Vol. iii. Philadelphia: J. B. Lippincott & Co. London: 16 Southampton Street, Covent Garden. 1883.

This is a volume of between seven and eight hundred pages, containing over five hundred illustrations. The fourteen chapters are upon the following subjects: Surgical Diseases of the Larynx and Trachea; Diseases and Injuries of the Nose and Naso-Pharyngeal Region, and the associated parts; Diseases and Injuries of the Eye and its Appendages; Diseases of the Ear; Malformations and Deformities; Tenotomy in the Treatment of Orthopædia; Affections of Muscles, Tendons, Bursæ and Aponeuroses; Surgical Affections of the Nerves; Surgical Affections of the Lymphatic System, Skin and Subcutaneous Connective Tissue; Syphilis; Tumors; Diseases of the Mammary Gland; Electricity in its Application to Surgical Therapeutics; Operations for Nerve Stretching and Massage. This volume concludes the work at which the author has been engaged for a period of five years. The illustrations are in great part original. It is a beautiful work, and needs but to be seen to confirm the high estimate so generally entertained regarding the author. The illustrations are especially interesting and valuable, for the reason that the vast majority are original and exceedingly well chosen. They illustrate many points in Pathology and Anatomy, in a manner such as we have scarcely, if ever, seen elsewhere. The text, also, is lucid, concise and to the point, showing the work to be the digest of the writer's mind and not simply a compilation and storehouse of the work and thought of others. To all this must be added the great advantage of being well up to date, and including the most recent discoveries and advances.

